# Multidimensional determinants of food insecurity among households in Somalia: evidence from the 2018/2019 Somalia health and demographic survey

**DOI:** 10.3389/fnut.2026.1845277

**Published:** 2026-06-18

**Authors:** Hassan Abdi Ahmed, Nelson Jerry Ndifwa

**Affiliations:** 1 Center for Graduate Studies, Department of Statistics and Data Analytics, Jamhuriya University of Science & Technology, Mogadishu, Somalia; 2 Eastern Africa Statistical Training Centre, Dar es Salaam, Tanzania

**Keywords:** food insecurity, households, nomads, rural populations, Somalia

## Abstract

**Background:**

Food insecurity remains a critical public health and development challenge in Somalia, particularly among rural and nomadic households, who are vulnerable to climate shocks, poor infrastructure, and socioeconomic constraints. Evidence on its scale and determinants is needed to guide targeted interventions.

**Objectives:**

To estimate the prevalence of food insecurity and associated multidimensional determinants among rural and nomadic households in Somalia.

**Methods:**

This study analyzed 9,399 rural and nomadic households from the 2018/2019 Somalia Health and Demographic Survey (SHDS) using the Food Insecurity Experience Scale (FIES). Multilevel ordinal logistic regression was applied to examine associated factors.

**Results:**

Overall, 58.86% of households were food secure or mildly food insecure, 9.13% experienced moderate food insecurity, and 32.01% experienced severe food insecurity. Larger household size was associated with higher odds of severe food insecurity (4–6 members: AOR = 1.22, 95% CI: 1.10–1.37, *p* < 0.01; ≥7 members: AOR = 1.47, 95% CI: 1.29–1.68, *p* < 0.01). Higher education was protective (AOR = 0.65, 95% CI: 0.54–0.78, *p* < 0.01). Lack of livestock (AOR = 1.37, 95% CI: 1.21–1.56, *p* < 0.01) and lack of electricity (AOR = 1.50, 95% CI: 1.20–1.86, *p* < 0.01) were associated with increased risk. Wealth showed a strong protective gradient, with progressively lower odds across higher quintiles (highest: AOR = 0.39, 95% CI: 0.28–0.53, *p* < 0.01). Nomadic households had lower odds of severe food insecurity than rural households (AOR = 0.78, 95% CI: 0.70–0.87, *p* < 0.01). Significant regional disparities were observed, with higher odds in Central (AOR = 2.45, *p* < 0.01) and Southern (AOR = 4.80, *p* < 0.01) regions compared to the North.

**Conclusion:**

Food insecurity in Somalia is shaped by socioeconomic, livelihood, and geographic factors. Targeted social protection, livelihood strengthening, and improved access to basic services are essential to reduce inequalities.

## Introduction

Food insecurity is defined as a condition in which individuals or households lack consistent physical and economic access to sufficient, safe, and nutritious food required for an active and healthy life (1–3). It is a multidimensional phenomenon influenced by the interaction of food availability, access, utilization, and stability within agrifood systems. Globally, food insecurity continues to affect a large proportion of the population. it remains one of the most pressing public health and development challenges, reflecting structural inequalities that limit people’s ability to obtain adequate and nutritious diets (4). In 2022, approximately 2.4 billion people (29.6%) experienced moderate to severe food insecurity, while 11.3% faced severe food insecurity (5). In addition, over 2 billion people suffer from micronutrient deficiencies, highlighting that food insecurity extends beyond hunger to include poor dietary (6). The COVID-19 pandemic, conflicts such as the Ukraine war, and rising food and energy prices have further intensified global hunger, with an estimated additional 50 million people pushed into food insecurity (5, 7, 8).

According to the United Nations World Population Prospects, the global population is projected to increase from approximately 8 billion in 2025 to 10.5 billion by 2100 (9). Rapid population growth intensifies the challenge of feeding more people with the planet’s limited resources, making it essential to improve food production efficiency, use natural resources sustainably, and reduce and reuse food and agricultural waste (10). In Canada, household food insecurity affected an estimated 11.6% of households in 2021, with 4.2% experiencing severe insecurity, where members reduced meal sizes or skipped meals (11). Approximately 2.33 billion people worldwide (28.9%) experienced moderate to severe food insecurity, with over 864 million (10.7%) facing severe food insecurity (12). In peri-urban areas, 29.9% of people were moderately or severely food insecure, compared to 25.5% in urban areas (12). Similarly, despite global progress in some regions, Africa, Western Asia, and parts of the Caribbean continue to experience worsening food insecurity due to economic shocks and climate-related stresses (13).

Regionally, the burden is disproportionately concentrated in low- and middle-income countries. Africa bears a disproportionate burden, with 58% of its population affected, nearly double the global average (12). Sub-Saharan Africa remains the most affected region, with approximately one in four people experiencing undernourishment (14). East Africa alone accounts for about 313 million people facing moderate to severe food insecurity, with 24.2% experiencing severe forms. In Ethiopia, recurrent drought, conflict, and inflation have left nearly 23 million people in need of food assistance, with urban food insecurity ranging from 41.3% to 92.4% (15, 16).

Somalia remains among the most food-insecure countries in the region, with persistent vulnerability driven by prolonged conflict, recurrent climate shocks, weak market systems, and limited access to essential services (17, 18). These structural constraints have weakened household resilience and disproportionately affected vulnerable groups, including pastoralists, agropastoralists, internally displaced persons (IDPs), women, and children (19). Although some progress has been made in governance and stabilization, improvements in food security outcomes remain limited due to persistent weaknesses in food availability, access, utilization, and affordability, thereby constraining progress toward national and global targets such as Somalia’s National Transformation Plan and Sustainable Development Goal 2 (Zero Hunger) (17, 18). In addition, approximately 68% of existing assessments still rely on poverty-based proxies rather than multidimensional vulnerability measures (20), limiting the accuracy and comprehensiveness of current evidence.

Despite ongoing efforts to address food insecurity, Somalia continues to experience high and persistent levels of vulnerability driven by interconnected shocks, including prolonged conflict, recurrent climate variability, market disruptions, and weak service delivery systems. These challenges have disproportionately affected rural, nomadic, and displaced populations, whose livelihoods are highly sensitive to environmental and economic shocks. As a result, food insecurity remains widespread, with limited improvements in resilience and household food access across different livelihood groups. However, a key research gap persists in the way food insecurity is measured and analyzed in the Somali context. In addition, most studies are descriptive in nature and provide limited analytical insight into how individual- and community-level factors jointly influence food insecurity outcomes. There is also a lack of studies that apply the Food Insecurity Experience Scale (FIES) within a multilevel modeling framework, particularly among nomadic and rural populations who are often underrepresented in national analyses. This gap constrains the development of targeted, evidence-based interventions. To address this gap, this study applies the FIES within a multilevel modeling framework to generate more precise and context-specific evidence on the determinants of food insecurity among nomadic and rural households in Somalia, thereby contributing novel empirical insights for targeted policy and intervention design. Therefore, the objective of this study is to examine the multidimensional determinants of food insecurity at both individual and community levels using data from the 2018/2019 Somalia Health and Demographic Survey, thereby contributing novel empirical insights to inform targeted policy and intervention design.

## Materials and methods

### Data source and design

This study employed a cross-sectional study using secondary data from the 2018/2019 Somalia Health and Demographic Survey (SHDS). The analysis examined household food insecurity in Somalia, alongside individual- and community-level determinants. The SHDS is the first nationally representative survey designed to collect comprehensive demographic and health-related information, including indicators of household food insecurity and key sociodemographic characteristics, conducted by the National Bureau of Statistics (NBS) in collaboration with the Ministry of Health and Human Services in Somalia (20, 21). The data were obtained from the household dataset available in the NBS microdata repository and were cleaned and processed as necessary for analysis. These data are essential for informing national and sub-national evidence-based policymaking, supporting planning and service delivery, and strengthening monitoring and evaluation of development programs (20).

### Target population and sample size

The Somalia Health and Demographic Survey applied a multi-stage stratified cluster sampling technique to ensure national representativeness, with stratification conducted by the National Bureau of Statistics (NBS) based on region and place of residence (urban, rural, and nomadic) (20). The original dataset comprised 18,823 household respondents. From this, rural and nomadic households were identified, yielding a subsample of 10,911 households. Subsequently, observations with missing Food Insecurity Experience Scale (FIES) responses (*n* = 1,512) were excluded, resulting in a final analytic sample of 9,399 households. The target population for this study includes all households residing in rural and nomadic areas with complete information on food insecurity, as measured by the FIES, and relevant sociodemographic variables. Accordingly, the final study sample consisted of 9,399 households drawn from the two strata (rural and nomadic).

### Study variables

Drawing on the existing body of literature, this study examined selected sociodemographic and geographical variables to assess the magnitude of food insecurity among rural and nomadic households, as well as to identify its key determinants and associated factors (3, 13, 14, 22).

### Outcome and explanatory variables

The dependent variable in this study was food insecurity, constructed using Food Insecurity Experience Scale (FIES) items, which assess households’ experiences during the preceding 30 days, including: not having enough food, eating smaller amounts of food, consuming fewer meals per day, experiencing a lack of food, going to sleep hungry, and spending an entire day without food. In the original survey data, households that provided an affirmative response to a particular Food Insecurity Experience Scale (FIES) item were coded as “Yes = 1” for that item, whereas households with no affirmative response were coded as “No = 0.” The responses to the eight FIES items were then aggregated to obtain a total food insecurity score for each household. Following the standard classification of the Food and Agriculture Organization (FAO), households were categorized into ordinal levels of food insecurity based on the severity of affirmative responses as follows: scores of 0–3 were classified as Food Secure/Mild Food Insecurity (0), scores of 4–6 as Moderate Food Insecurity (1), and scores of 7–8 as Severe Food Insecurity (2) (13, 23). This classification facilitated the application of multilevel ordinal logistic regression analysis. This classification facilitated the application of multilevel ordinal logistic regression analysis. The explanatory variables were classified into three content-focused categories: (i) Individual-level characteristics: age of household head (24), sex of household head (25, 26), educational status of household members (14, 27), head’s marital status (25, 26), household size (27, 28), agricultural land ownership (3, 29), livestock ownership, bank account ownership (30), livestock loss in the past year (31) and school attendance (14, 32), (ii) Household level determinants wealth index (2), access to electricity and (iii) Community-level determinants: and geographical factors like type of residence of the households (33) and region (34).

### Inclusion and exclusion criteria

**FIGURE 1:**
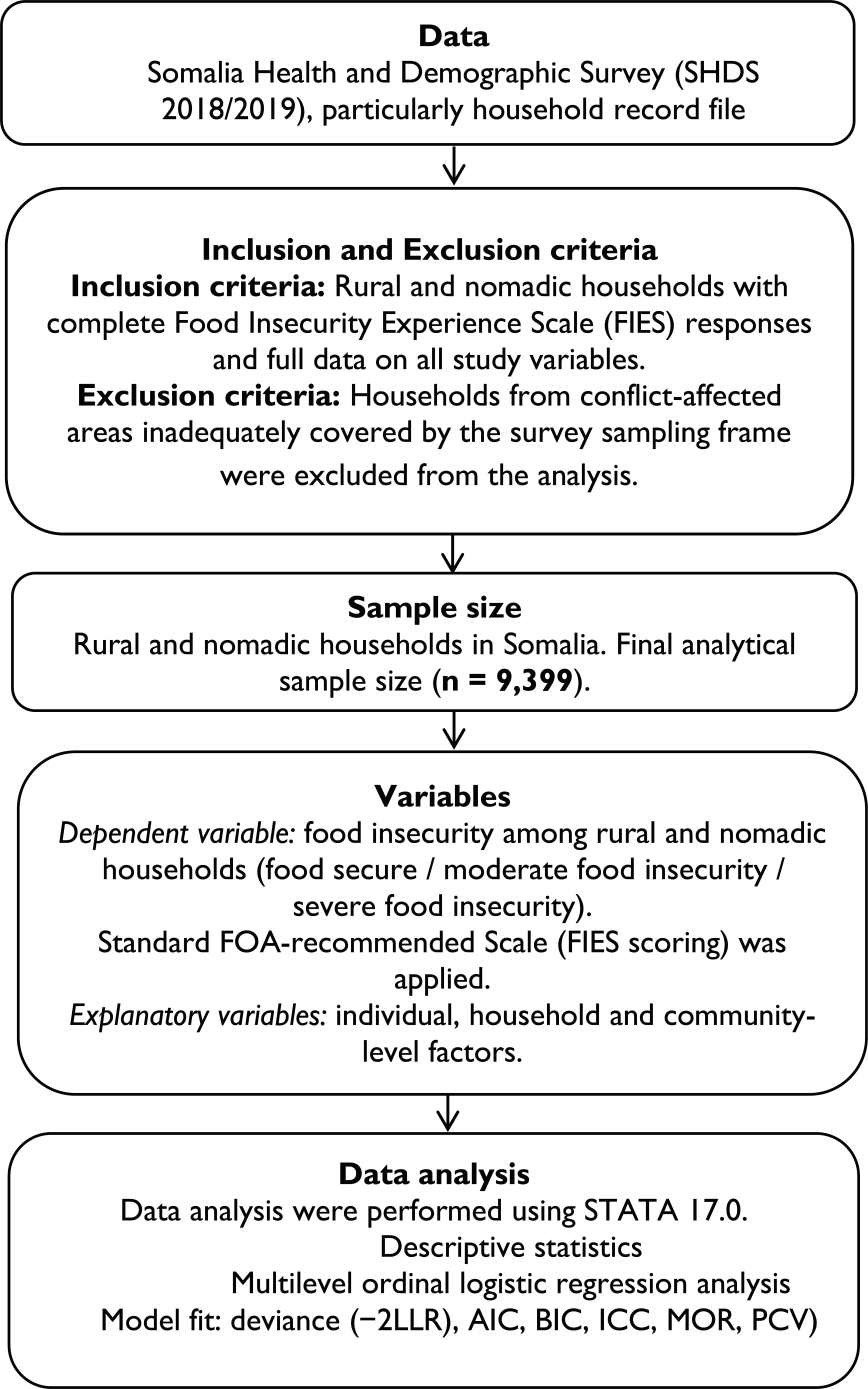
Study selection flowchart developed by the authors.

The target population for this study comprised households residing in rural and nomadic areas of Somalia captured in the 2018/2019 Somalia Health and Demographic Survey. Households were included if they: (i) belonged to rural or nomadic population categories, (ii) had complete data on the Food Insecurity Experience Scale (FIES). Households from conflict-affected areas inadequately covered by the survey sampling frame were excluded from the analysis. The study selection process, including the data source, eligibility criteria, final analytical sample, variable classification, and statistical analyses, is presented in Figure 1.

### Method of analysis

The unit of analysis was constituted for all rural and nomadic households in Somalia. Initially, to ensure methodological rigor, the complex survey design of the 2018/2019 Somalia Health and Demographic Survey (SHDS) was accounted for in all analyses using the svyset procedure. Sampling weights (wgt) were applied to adjust for unequal probabilities of selection, ensuring nationally representative estimates. The multi-stage sampling design was incorporated by specifying the Enumeration Area (EA) as the primary sampling unit (PSU) to appropriately reflect data clustering. Variance estimation was conducted using a design-based covariance matrix to produce robust standard errors that accurately accounted for the survey design. Subsequently, descriptive statistics, including frequencies and percentages, were computed to summarize the characteristics of the study population. Subsequently, Pearson’s chi-square test of independence was conducted to assess associations between the outcome variable (food insecurity) and the explanatory factors. To ensure robustness of the study, multicollinearity among independent variables was evaluated using the Variance Inflation Factor (VIF), and no evidence of multicollinearity was observed, as all VIF values were below the acceptable threshold of 5. Variables with a *p*-value < 0.25 in the chi-square analyses were considered eligible and were entered into the multilevel ordinal regression model. All selected variables met the inclusion criteria. In the multilevel analysis, adjusted odds ratios (AORs) with 95% confidence intervals (CIs) were estimated, and statistical significance was established at *p* < 0.05. All statistical analyses were conducted using Stata version 17. In this analysis, four hierarchical models were estimated to identify the specification that most appropriately explained the data. The initial model (Model I) was the null model, which contained no explanatory variables and was used to quantify the proportion of variation in food insecurity attributable to clustering effects. Model II incorporated only individual-level covariates, Model III included only community-level covariates, and Model IV combined both individual- and community-level predictors.

### Model development, random-effects analysis, and selection of the best-fitted model

To quantify the extent of clustering and between-cluster variation in food insecurity among rural and nomadic households in Somalia, three complementary measures of random effects were estimated: The Intraclass Correlation Coefficient (ICC), the Proportional Change in Variance (PCV), and the Median Odds Ratio (MOR). These measures were used to assess the magnitude of contextual (community-level) effects and the extent to which observed covariates explain between-cluster heterogeneity. The ICC estimates the proportion of total variation in food insecurity attributable to differences between clusters. A MOR greater than 1 indicates the presence of meaningful contextual variation, with higher values reflecting stronger between-cluster inequality. The PCV was estimated to assess the proportion of cluster-level variance explained by the inclusion of covariates across successive models, and higher PCV values indicate greater explanatory power of the included covariates in reducing between-cluster variation in food insecurity. Model evaluation and selection were guided by log-likelihood ratio (LLR), deviance statistics (−2LL), the Akaike Information Criterion (AIC) and Bayesian information Criterion (BIC). The preferred model was identified as the one with the highest log-likelihood and the lowest deviance and AIC values. Overall, the goodness-of-fit statistics demonstrated that the inclusion of individual- and community-level variables markedly enhanced model performance relative to the null model. Progressive improvements were observed across models, as reflected by increasing Deviance (−2LL) values and corresponding reductions in deviance, AIC, and BIC values. The lowest values for these criteria were observed in the full model (Model IV), indicating that the combined model provided the most comprehensive explanation of food insecurity.

## Results of the study

### Characteristics of the respondents across demographic, socioeconomic, and geographic variables

#### Individual-level determinants

Table 1 presents the demographic and socioeconomic characteristics of the study population. A total of 9,399 households were included in the analysis. At the individual level, household heads were predominantly male 66.83%, with female-headed households accounting for 33.17%. Most household heads were aged 30–64 years 68.77%, followed by those aged 13–29 years 18.34%, while 12.88% were aged 65 years or older. In terms of household size, 44.00% of households comprised 4–6 members, 32.39% had seven or more members, and 23.61% had 1–3 members. Educational attainment was low, with 89.68% of households reporting uneducated members and 10.32% having members with primary or higher education. Agricultural land ownership was reported by 20.44% of households, while 79.56% did not own land. Livestock ownership was widespread, 76.56%; however, more than half of households experienced livestock loss in the past year, 53.06%. Access to formal financial services was extremely limited, with only 0.57% of households owning a bank account. School attendance was reported by 26.58% of households, whereas 73.42% reported no school attendance. Regarding marital status, the majority of household heads were married (83.18%), followed by widowed 9.63%, divorced 3.99%, abandoned 1.59%, and never married 1.62%.

**TABLE 1:** Characteristics of the respondents across demographic, socioeconomic, and geographic variables (*n* = 9,399).

Individual-level variable	Frequency	Percent
Household size		
1–3 members	2,219	23.61
4–6 members	4,136	44.00
≥7 members	3,044	32.39
Age of household head		
13–29 years	1,724	18.34
30–64 years	6,464	68.77
65+ years	1,211	12.88
Sex of the household head		
Male	6,281	66.83
Female	3,118	33.17
Educational status of household members		
Uneducated	8,429	89.68
Educated	970	10.32
Agricultural land ownership		
Yes	1,921	20.44
No	7,476	79.56
Livestock ownership		
Yes	7,195	76.56
No	2,203	23.44
Bank account ownership		
Yes	54	0.57
No	9,343	99.43
Livestock loss in the past year		
Yes	4,987	53.06
No	4,411	46.94
Household head’s marital status		
Married	7,818	83.18
Divorced	375	3.99
Abandoned	149	1.59
Widowed	905	9.63
Never married	152	1.62
School attendance		
Yes	2,498	26.58
No	6,900	73.42
Household-level factors		
**Wealth index**		
Lowest	5,808	61.79
Second	1,474	15.68
Middle	1,126	11.98
Fourth	648	6.89
Highest	343	3.65
Access to electricity		
Yes	792	8.43
No	8,607	91.57
Community-level variable		
**Type of residence**		
Rural	4,484	47.71
Nomadic	4,915	52.29
Region of residence		
Northern	5,441	57.89
Central	2,292	24.39
Southern	1,666	17.73

#### Household-level determinants

Most households were classified in the lowest wealth quintile, 61.79%, with smaller proportions in the second, 15.68%, middle, 11.98%, fourth, 6.89%, and highest, 3.65% quintiles. Access to electricity was very limited, with only 8.43% of households reporting access, compared to 91.57% without electricity.

#### Community-level determinants

At the community level, slightly more than half of households resided in nomadic settings 52.29%, while 47.71% lived in rural areas. Regionally, the majority of households were located in the Northern region 57.89%, followed by the Central 24.39%, and Southern 17.73% regions.

### Prevalence of food insecurity among rural and nomadic households in Somalia

Table 2 presents the prevalence of food insecurity among rural and nomadic households in Somalia. Notably, nearly one-third of households (32.01%) experienced severe food insecurity, representing the most critical finding, while moderate food insecurity was comparatively lower at 9.13%.

**TABLE 2:** Prevalence of food insecurity among rural and nomadic households in Somalia (*n* = 9,399).

Food insecurity category	Prevalence	Std. error	95% CI
Food secure/mild food insecurity	58.86%	0.0051	0.5786, 0.5985
Moderate food insecurity	9.13%	0.0030	0.0856, 0.0973
Severe food insecurity	32.01%	0.0048	0.3108, 0.3296

### Household food insecurity in rural and nomadic areas in Somalia

**FIGURE 2:**
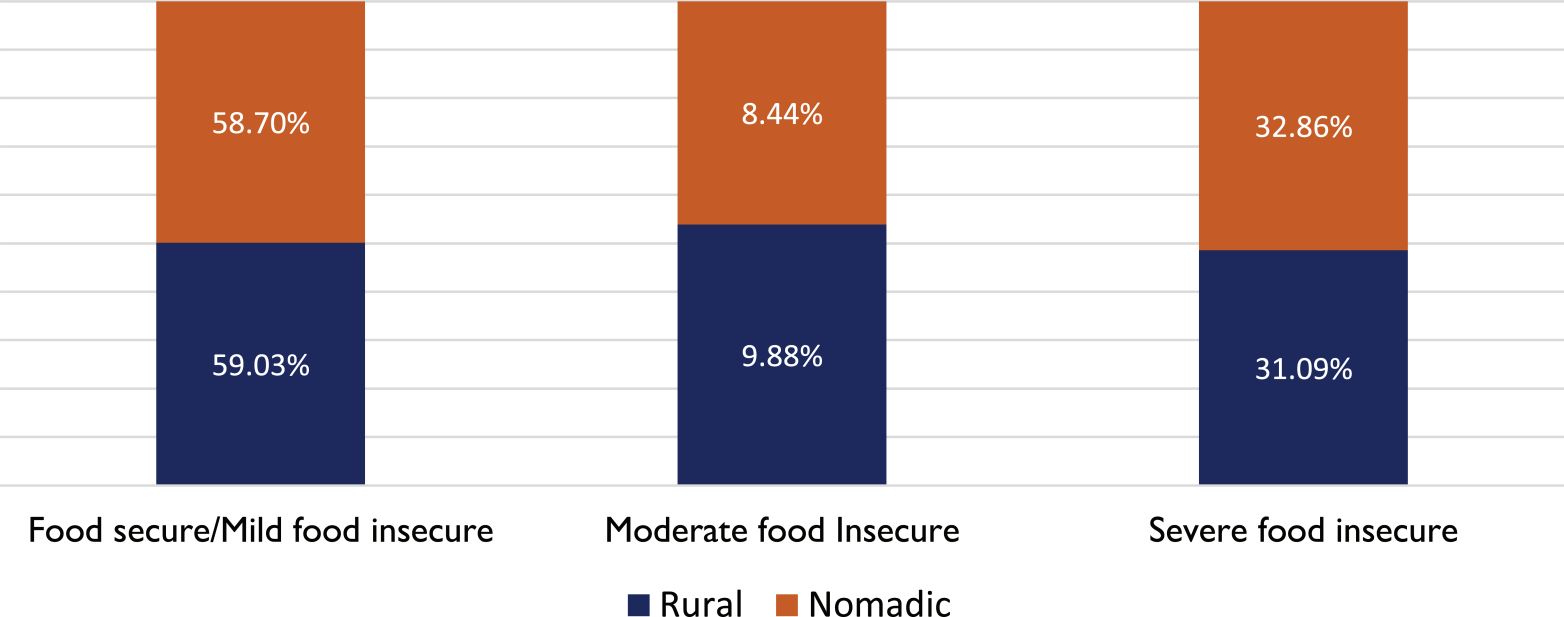
Household food insecurity in rural and nomadic areas in Somalia (*n* = 9,399).

Figure 2 shows the distribution of household food insecurity among rural and nomadic areas. Among rural households, 59.03% were food secure or mildly food insecure, 9.88% experienced moderate food insecurity, and 31.09% experienced severe food insecurity. Similarly, among nomadic households, 58.70% were food secure or mildly food insecure, 8.44% experienced moderate food insecurity, and 32.86% experienced severe food insecurity. The findings indicate that severe food insecurity was slightly higher among nomadic households compared to rural households.

### Association between food insecurity and household characteristics

Household food insecurity was significantly associated with all examined household and community-level factors at *p* < 0.025 among rural and nomadic households in Somalia. These included household size (χ^2^ = 19.85), sex of household head (χ^2^ = 20.78), education of household members (χ^2^ = 69.60), marital status (χ^2^ = 43.56), agricultural land ownership (χ^2^ = 103.46), livestock ownership (χ^2^ = 13.06), family bank account ownership (χ^2^ = 6.75), livestock loss in the last year (χ^2^ = 114.29), wealth index (χ^2^ = 187.94), access to electricity (χ^2^ = 140.28), type of residence (χ^2^ = 7.64), and region of residence (χ^2^ = 842.99), all at a significance level of *p* < 0.05 (Table 3).

**TABLE 3:** Association between food insecurity and household characteristics (*n* = 9,399).

Individual-level factors	Food secure		Moderate food insecure		Severe food insecure		Pearson chi-square test	
	*n*	%	*n*	%	*n*	%	χ^2^	*P*-value
Household size							19.85	*P* < 0.001
1–3 members	1,378	62.10	186	8.38	655	29.52	–	–
4–6 members	2,448	59.19	378	9.14	1,310	31.67	–	–
≥7 members	1,706	56.04	294	9.66	1,044	34.30	–	–
Sex of household head							20.78	*P* < 0.001
Male	3,799	60.48	547	8.71	1,935	30.81	–	–
Female	1,733	55.58	311	9.97	1,074	34.45	–	–
Education of household members							69.60	*P* < 0.001
Uneducated	4,850	57.54	768	9.11	2,811	33.35	–	–
Educated	682	70.31	90	9.28	198	20.41	–	–
Marital status							43.56	*P* < 0.001
Married	4,677	59.82	696	8.90	2,445	31.27	–	–
Divorced	203	54.13	42	11.20	130	34.67	–	–
Abandoned	74	49.66	13	8.72	62	41.61	–	–
Widowed	469	51.82	94	10.39	342	37.79	–	–
Never married	109	71.71	13	8.55	30	19.74	–	–
Agricultural land ownership							103.46	*P* < 0.001
Yes	935	48.67	214	11.14	772	40.19	–	–
No	4,595	61.46	644	8.61	2,237	29.92	–	–
Livestock ownership							13.06	*P* < 0.001
Yes	4,182	58.12	641	8.91	2,372	32.97	–	–
No	1,349	61.23	217	9.85	637	28.91	–	–
Bank account ownership							6.75	*P* < 0.001
Yes	41	75.93	2	3.70	11	20.37	–	–
No	5,489	58.75	856	9.16	2,998	32.09	–	–
Livestock loss							114.29	*P* < 0.001
Yes	2,756	55.26	397	7.96	1,834	36.78	–	–
No	2,775	62.91	461	10.45	1,175	26.64	–	–
School attendance							20.58	*P* < 0.001
Yes	1,436	57.49	284	11.37	778	31.14	–	–
No	4,095	59.35	574	8.32	2,231	32.33	–	–
Household-level factors								
Wealth index							187.94	*P* < 0.001
Lowest	3,242	55.82	531	9.14	2,035	35.04	–	–
Second	822	55.77	144	9.77	508	34.46	–	–
Middle	710	63.06	109	9.68	307	27.26	–	–
Fourth	472	72.84	52	8.02	124	19.14	–	–
Highest	286	83.38	22	6.41	35	10.20	–	–
Access to electricity							140.28	*P* < 0.001
Yes	620	78.28	55	6.94	117	14.77	–	–
No	4,912	57.07	803	9.33	2,892	33.60	–	–
Community-level factors								
Type of residence							7.64	*P* = 0.022
Rural	2,647	59.03	443	9.88	1,394	31.09	–	–
Nomadic	2,885	58.70	415	8.44	1,615	32.86	–	–
Region							842.99	*P* < 0.001
Northern	3,826	70.32	384	7.06	1,231	22.62	–	–
Central	1,144	49.91	288	12.57	860	37.52	–	–
Southern	562	33.73	186	11.16	918	55.10	–	–

### Prevalence of food insecurity by household electricity access in Somalia

**FIGURE 3:**
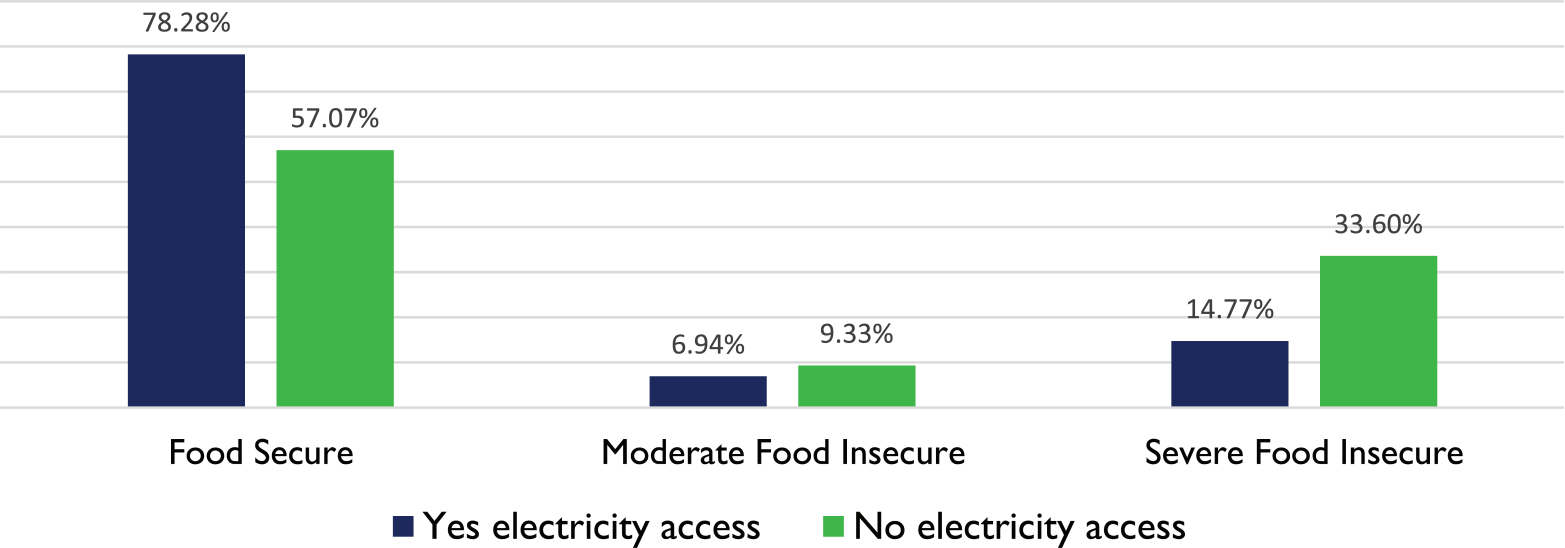
Prevalence of food insecurity by household electricity access in Somalia (*n* = 9,399).

Figure 3 presents the distribution of food insecurity status by household electricity access. Among households with electricity access, 78.28% were food secure, 6.94% experienced moderate food insecurity, and 14.77% experienced severe food insecurity. In contrast, among households without electricity access, 57.07% were food secure, 9.33% experienced moderate food insecurity, and 33.60% experienced severe food insecurity. The figure indicates a substantially higher prevalence of severe food insecurity among households without electricity access compared to those with electricity access.

### Multilevel ordinal logistic regression analysis

#### Multilevel multivariable ordinal logistic regression analysis of individual- and community-level determinants of household food insecurity

Table 4 presents results from a multilevel ordinal logistic regression model examining individual- and community-level determinants of household food insecurity in Somalia. The model estimates the likelihood of households being in higher categories of food insecurity (from food secure to moderate and severe food insecurity), accounting for clustering at the residence level. At the individual level, household size was significantly associated with increased severity of food insecurity. Compared with smaller households (1–3 members), households with 4–6 members (AOR = 1.22, 95% CI: 1.10–1.37) and those with seven or more members (AOR = 1.47, 95% CI: 1.29–1.68) had higher odds of being in more severe food insecurity categories, indicating that larger households are more vulnerable. Education of household members showed a protective effect, as households with primary or higher education were less likely to experience severe food insecurity compared to uneducated households (AOR = 0.65, 95% CI: 0.54–0.78). Asset ownership was also an important determinant. Households without agricultural land had 16% lower odds of severe food insecurity than land-owning households (AOR = 0.84; 95% CI: 0.74–0.93), whereas livestock ownership was associated with increased vulnerability when absent (AOR = 1.37; 95% CI: 1.21–1.56). In contrast, households that did not experience livestock loss were significantly less likely to fall into more severe food insecurity categories (AOR = 0.55, 95% CI: 0.50–0.61), highlighting the importance of livestock shocks. Similarly, School attendance was significantly associated with food insecurity; households with school attendance had 17% lower odds of severe food insecurity (AOR = 0.83; 95% CI: 0.73–0.93). Age of the household head showed a modest effect, where households headed by individuals aged 65 years and above had higher odds of more severe food insecurity (AOR = 1.19, 95% CI: 1.01–1.41). Sex of household head and bank account ownership were not statistically significant predictors. In contrast, at the household level, the wealth index showed a strong, consistent protective gradient. Compared to the poorest households, those in higher wealth quintiles had progressively lower odds of severe food insecurity: second (AOR = 0.81), middle (AOR = 0.79), fourth (AOR = 0.65), and highest (AOR = 0.39), all statistically significant. Similarly, access to electricity was also significant, with households lacking electricity having higher odds of severe food insecurity (AOR = 1.50, 95% CI: 1.20–1.86). At the community level, residence type showed that nomadic households were less likely to experience severe food insecurity compared to rural households (AOR = 0.78, 95% CI: 0.70–0.87). Strong regional disparities were also observed, with households in the Central region (AOR = 2.45, 95% CI: 2.20–2.74) and Southern region (AOR = 4.80, 95% CI: 4.22–5.45) showing substantially higher odds of severe food insecurity compared to those in the Northern region. Overall, the model confirms that both individual- and community-level factors significantly shape the severity of household food insecurity, with clear evidence of socioeconomic and geographic inequalities.

**TABLE 4:** Multilevel ordinal logistic regression analysis of individual- and community-level determinants of household food insecurity (*n* = 9,399).

Individual-level variable	Model I (null model)	Model II (individual-level factors)	Model III (community-level factors)	Model IV (individual and community factors)
Household size		AOR (95% CI)		AOR (95% CI)
1–3 members	–	1	–	1
4–6 members	–	1.22 (1.06–1.35)*	–	1.22 (1.10–1.37)*
≥7 members	–	1.46 (1.24–1.67)*	–	1.47 (1.29–1.68)*
Age of household head				
13–29 years	–	1	–	1
30–64 years	–	0.98 (0.87–1.11)	–	0.99 (0.88–1.12)
65+ years	–	1.18 (1.00–1.40)*	–	1.19 (1.01–1.41)*
Sex of household head				
Male	–	1	–	1
Female	–	1.08 (0.97–1.21)	–	1.07 (0.96–1.20)
Education of household members				
Uneducated	–	1	–	1
Primary and higher	–	0.64 (0.53–0.77)*	–	0.65 (0.54–0.78)*
Agricultural land ownership				
Yes	–	1	–	1
No	–	0.83 (0.74–0.93)*	–	0.84 (0.75–0.94)*
Livestock ownership				
Yes	–	1	–	1
No	–	1.36 (1.20–1.55)*	–	1.37 (1.21–1.56)*
Bank account ownership				
Yes	–	1	–	1
No	–	1.07 (0.92–1.25)	–	1.08 (0.93–1.26)
Livestock loss				
Yes	–	1	–	1
No	–	0.54 (0.49–0.60)*	–	0.55 (0.50–0.61)*
School attendance				
Yes	–	0.82 (0.73–0.93)*	–	0.83 (0.74–0.94)*
No	–	1	–	1
Household-level factors				
Wealth index				
Lowest	–	–	1	1
Second	–	–	0.80 (0.70–0.92)*	0.81 (0.71–0.93)*
Middle	–	–	0.78 (0.67–0.91)*	0.79 (0.68–0.92)*
Fourth	–	–	0.64 (0.51–0.80)*	0.65 (0.52–0.81)*
Highest	–	–	0.38 (0.27–0.52)*	0.39 (0.28–0.53)*
Access to electricity				
Yes	–	–	1	1
No	–	–	1.51 (1.21–1.87)*	1.50 (1.20–1.86)*
Type of residence				
Rural	–	–	1	1
Nomadic	–	–	0.77 (0.69–0.86)*	0.78 (0.70–0.87)*
Region				
Northern	–	–	1	1
Central	–	–	2.44 (2.19–2.73)*	2.45 (2.20–2.74)*
Southern	–	–	4.79 (4.21–5.44)*	4.80 (4.22–5.45)*
Random-effects measures				
Variance	0.1430	0.1680	0.1220	0.0785
ICC	4.17%	4.86%	3.58%	2.33%
MOR	1.43	1.48	1.40	1.31
PCV	Reference	−17.5%	14.70%	45.10%
Model fit evaluation				
Log likelihood	−8413.34	−8144.45	−8006.64	−7778.87
AIC	16828.67	16338.90	16025.28	15591.74
BIC	16854.11	16499.30	16059.03	15795.60

IC, intra-cluster correlation; MOR, median odds ratio; PCV, proportional change in variance; LLR, log-likelihood ratio; AIC, Akaike Information Criterion; BIC, Bayesian Information Criterion; The asterisk (*) indicates statistical significance at *p* < 0.05 in the multilevel analysis.

#### Model comparison, goodness-of-fit, and random-effects analysis of determinants of food insecurity among rural and nomadic households

Random-effects analysis and model fit comparisons for the multilevel ordinal logistic regression models are presented (Table 4). To assess the appropriateness of the multilevel framework, a null (empty) model was first estimated. The null model revealed statistically significant between-cluster variation in food insecurity, with a random intercept variance of 0.1430 and a highly significant likelihood ratio test (*p* < 0.001). This confirms the presence of meaningful clustering at the community level and justifies the use of multilevel modeling. The intraclass correlation coefficient (ICC) further indicated that a non-negligible proportion of total variation in food insecurity was attributable to between-cluster differences. The ICC was 4.17% in the null model, increased slightly to 4.86% in Model I, and then declined to 3.58% in Model II and 2.33% in the fully adjusted Model III. This pattern suggests that while clustering remains present, the inclusion of covariates, particularly in the final model, helped to explain part of the between-cluster heterogeneity.

The median odds ratio (MOR) also confirmed the relevance of contextual effects. The MOR in the null model was 1.43, indicating that moving from a lower-risk to a higher-risk cluster increases the median odds of food insecurity by 43% for otherwise identical households. The MOR slightly increased in Model I (1.48), then declined in Model II (1.40), and further reduced to 1.31 in Model III, reflecting a gradual reduction in unexplained regional heterogeneity after adjustment for individual- and community-level factors. The proportional change in variance (PCV) provides additional insight into the explanatory contribution of successive models. Model I showed a negative PCV (−17.5%), indicating that the inclusion of individual-level covariates alone did not reduce between-cluster variance and instead slightly increased unexplained heterogeneity. In contrast, Model II showed a positive PCV (14.7%), suggesting that community-level characteristics contributed to explaining regional variation. The fully adjusted Model III achieved the highest PCV (45.1%), indicating that combined individual- and community-level factors explained nearly half of the initial between-cluster variation in food insecurity, demonstrating substantially improved explanatory power.

Model fit statistics further supported the superiority of the fully adjusted model. Model comparison using deviance (−2 log-likelihood), Akaike Information Criterion (AIC), and Bayesian Information Criterion (BIC) consistently indicated that Model III provided the best fit to the data. Specifically, Model III recorded the lowest deviance (15,557.74), AIC (15,591.74), and BIC (15,795.60), indicating the most parsimonious and best-fitting representation among all competing models. Overall, these findings demonstrate that food insecurity among rural and nomadic households in Somalia is shaped by both individual and contextual factors, with persistent but partially explained clustering at the community level. The fully adjusted model provides the most robust and comprehensive explanation of variability in food insecurity.

## Discussion

This study explored multidimensional determinants of food insecurity among rural and nomadic households in Somalia. The findings indicated that 41.14% of households were food insecure at the time of the survey. Among these, 9.13% experienced moderate food insecurity, while 32.01% experienced severe food insecurity. This reflects a substantial burden of food insecurity among rural and nomadic households. The observed prevalence is consistent with findings reported in similar settings, including Southern Ethiopia (42.2%) (35), Somalia (45.7%) (14), Eastern Ethiopia (41.7%) (26, 31), and Northern Ethiopia (approximately 40%–43%) (36).

Estimates from the multilevel ordinal logistic regression model indicate that household size was positively associated with food insecurity. Specifically, households with 4–6 members (AOR: 1.22) had higher odds of food insecurity compared to households with 1–3 members, while those with ≥7 members (AOR: 1.47) were even more likely to experience food insecurity. This finding is consistent with previous studies in Ethiopia (24, 37–39). The observed relationship may be explained by increased household consumption needs and limited resource availability in larger households, which heightens vulnerability to food shortages. Similarly, education emerged as a protective factor against food insecurity. Household members with at least a primary education (AOR: 0.65) had lower odds of food insecurity compared to uneducated households. Education likely enhances household decision-making, income-generating opportunities, and adoption of improved livelihood strategies, thereby improving food security outcomes. This finding aligns with evidence from Ethiopia and other settings, where higher education is consistently associated with reduced vulnerability to food insecurity (31, 37, 40).

In contrast, agricultural land ownership was also significantly associated with food insecurity. Households without agricultural land had 16% lower odds of severe food insecurity compared to land-owning households (AOR = 0.83; 95% CI: 0.74–0.93). Similar findings have been reported in Ethiopia and Madagascar (35, 41). This unexpected finding may reflect the Somali context, where ownership of agricultural land does not necessarily guarantee stable food production due to recurrent droughts, conflict, displacement, and limited agricultural infrastructure. In addition, households without land may rely more on diversified livelihood strategies, such as petty trade, remittances, livestock activities, or humanitarian assistance, which may provide more flexible sources of income and food access during periods of environmental and economic instability. Likewise, livestock ownership showed a significant association with food insecurity. Households without livestock had higher odds of food insecurity (AOR: 1.37) compared to livestock-owning households. This finding is consistent with previous studies (3, 31, 42–44). Livestock plays a crucial role in providing food, income, and financial resilience, acting as a key buffer against shocks in pastoral and agro-pastoral systems.

Similarly, households that did not experience livestock loss had lower odds of food insecurity (AOR: 0.55), indicating that livestock shocks significantly increase vulnerability. This is supported by evidence from previous studies highlighting livestock loss as a major driver of household food insecurity in pastoral contexts (45–47). Livestock functions as both a productive asset and a coping mechanism; therefore, its loss directly undermines household food access and resilience. However, households headed by older individuals (65 years and above) had higher odds of food insecurity (AOR: 1.19) compared to younger heads. This finding is consistent with previous studies showing that older adults are particularly vulnerable to food insecurity due to declining physical capacity, limited income-generating opportunities, and inadequate social protection systems (14, 48). This finding may reflect reduced physical capacity and limited income-generating potential among elderly household heads, increasing their vulnerability to food insecurity and household food shortages.

In another perspective, households with school attendance had 17% lower odds of severe food insecurity (AOR = 0.82; 95% CI: 0.73–0.93), consistent with studies showing that education improves access to information, employment opportunities, and adaptive capacity (14, 32, 37). This finding may suggest that school attendance is associated with improved household welfare and access to resources. However, in the rural and nomadic Somali context, lower school attendance may also reflect children’s involvement in livestock herding and other livelihood-supporting activities that contribute directly to household food access and economic survival. In contrast, wealth status showed a strong graded protective effect. Compared with the poorest households, those in higher wealth quintiles had progressively lower odds of food insecurity: second (AOR: 0.81), middle (AOR: 0.79), fourth (AOR: 0.65), and highest (AOR: 0.39). This finding is consistent with extensive literature showing that poverty is a key driver of food insecurity, particularly in fragile and low-income settings (32, 49–53).

On the other hand, access to electricity was significantly associated with food insecurity. Households without electricity had higher odds of food insecurity, reflecting the role of infrastructure in improving livelihoods, food storage, and economic activities. Similar findings have been reported in other low-resource settings (54–56). This underscores the unique vulnerabilities of mobile populations and may reflect limited access to economic opportunities, food storage facilities, market information, and essential social services in areas lacking electricity infrastructure. Additionally, the type of residence was also significant. Nomadic households had lower odds of food insecurity compared to rural households (AOR: 0.78). This may reflect livelihood diversification and mobility patterns that enable pastoral households to adapt to environmental variability. However, evidence across contexts remains mixed, with some studies reporting higher vulnerability among nomadic populations due to service access constraints (14, 57). Differences in livelihood strategies may explain this association, as rural households often rely on agro-pastoral production systems that can provide more stable access to food and income compared with nomadic households, which are more exposed to environmental shocks and seasonal food scarcity. Finally, strong regional disparities were observed. Compared with the northern region, households in the central (AOR: 2.45) and southern regions (AOR: 4.80) had significantly higher odds of food insecurity. This likely reflects differences in conflict exposure, infrastructure, market access, and institutional stability, with southern and central Somalia experiencing more persistent insecurity and disruption to livelihoods.

In conclusion, this study highlights that food insecurity among rural and nomadic households in Somalia is driven by multiple interrelated household and contextual factors, including socioeconomic status, livelihood assets, infrastructure access, and regional disparities. The findings underscore the need for integrated and context-specific interventions that strengthen household resilience, improve access to essential services, and address structural inequalities, with particular attention to high-risk regions. Overall, targeted and multisectoral policy responses are essential to effectively reduce food insecurity in Somalia.

## Limitations of the study

Although this study provides important insights into the determinants of food insecurity in Somalia, several limitations should be acknowledged. First, the exclusion of conflict-affected populations may have introduced selection bias. Second, the cross-sectional design limited causal inference between key variables and food insecurity. Third, spatial displacement of survey cluster locations may have caused geographic misclassification, particularly in nomadic areas. Fourth, a key limitation of this study is that the analysis is based on 2018/2019 survey data, which do not capture the effects of more recent shocks, including the severe 2022–2023 drought and the 2024 floods that substantially affected food security conditions in Somalia. Consequently, the findings may not fully reflect the current magnitude and dynamics of food insecurity in the country. Finally, reliance on self-reported data may have introduced reporting bias and underestimated food deprivation.

### Policy implications

The findings indicate that food insecurity among rural and nomadic households in Somalia is shaped by a combination of individual and community-level determinants, requiring integrated and context-specific policy responses. At the individual level, larger households are more vulnerable to food insecurity, suggesting the need to strengthen and scale up social protection programs that prioritize households with higher dependency ratios. The protective effect of education and school attendance highlights the importance of investing in human capital through expanded access to education, school feeding initiatives, and complementary household support programs to enhance long-term resilience. Livelihood-related assets also play a critical role in food security outcomes. Households without livestock and those experiencing livestock losses are significantly more vulnerable, underscoring the need for livestock protection strategies, including drought preparedness interventions, and timely restocking programs following shocks. Similarly, improving access to financial services and strengthening livelihood diversification opportunities can help reduce household vulnerability. At the community level, substantial wealth disparities, limited access to electricity, and marked regional differences, particularly the higher vulnerability observed in Central and Southern Somalia, highlight the importance of geographically targeted interventions. Policy efforts should prioritize investment in rural infrastructure, energy access, market integration, and financial inclusion. Strengthening these structural determinants, while tailoring interventions to the dominant pastoral and agro-pastoral livelihood systems, is essential for sustainably reducing food insecurity across Somalia.

## Data Availability

The datasets presented in this study can be found in online repositories. The names of the repository/repositories and accession number(s) can be found below: https://microdata.nbs.gov.so/index.php/catalog/59/get-microdata.
